# iCAV: an integrative database of cancer-associated viruses

**DOI:** 10.1093/database/baab079

**Published:** 2021-12-15

**Authors:** Bo Liu, Qingfeng Zhang, Jingou Wang, Shumin Cao, Zhiyuan Zhou, Ze-Xian Liu, Han Cheng

**Affiliations:** School of Life Sciences, Zhengzhou University, Zhengzhou 450001, China; State Key Laboratory of Oncology in South China, Collaborative Innovation Center for Cancer Medicine, Sun Yat-sen University Cancer Center, Guangzhou 510060, China; School of Life Sciences, Zhengzhou University, Zhengzhou 450001, China; School of Life Sciences, Zhengzhou University, Zhengzhou 450001, China; School of Life Sciences, Zhengzhou University, Zhengzhou 450001, China; State Key Laboratory of Oncology in South China, Collaborative Innovation Center for Cancer Medicine, Sun Yat-sen University Cancer Center, Guangzhou 510060, China; School of Life Sciences, Zhengzhou University, Zhengzhou 450001, China

## Abstract

To date, various studies have found that the occurrence of cancer may be related to viral infections. Therefore, it is important to explore the relationship between viruses and diseases. The International Agency for Research on Cancer has defined six types of viruses as Class 1 human carcinogens, including Epstein–Barr virus, hepatitis C virus, hepatitis B virus, human T-cell lymphotropic virus, human herpesvirus 8 and human papillomavirus, while Merkel cell polyomavirus is classified as ‘probably carcinogenic to humans’ (Group 2A). Therefore, in-depth research on these viruses will help clarify their relationship with diseases, and substantial efforts have been made to sequence their genomes. However, there is no complete database documenting these cancer-associated viruses, and researchers are not able to easily access and retrieve the published genomes. In this study, we developed iCAV, a database that integrates the genomes of cancer-related viruses and the corresponding phenotypes. We collected a total of 18 649 genome sequences from seven human disease-related viruses, and each virus was further classified by the associated disease, sample and country. iCAV is a comprehensive resource of cancer-associated viruses that provides browse and download functions for viral genomes.

**Database URL**: http://icav.omicsbio.info/

## Introduction

Since the early 1900s, various studies have reported the carcinogenic properties of retroviruses (1). In the 1960s, Sir Anthony Epstein, Bert Achong and Yvonne Barr identified the first human tumor virus in a cell culture of samples from pediatric Burkitt’s lymphoma patients in Africa; that virus was named the Epstein–Barr virus (EBV) (2). Since then, evidence of the association between cancers and infections with certain viruses has been accumulating, and people have identified several cancer-associated viruses, including EBV, human papillomavirus (HPV), Kaposi’s sarcoma-associated herpesvirus (KSHV; also known as human herpesvirus 8, HHV8), hepatitis C virus (HCV), hepatitis B virus (HBV), Merkel cell polyomavirus (MCV) and human T-cell lymphotropic virus (HTLV) (3). Infection with these viruses is the etiology of approximately 15% of all cancer cases worldwide (4). According to the assessment of the International Agency for Research on Cancer (IARC), HBV and HCV are indirect carcinogens that cause cancers by promoting a chronic inflammatory state, while HPV, MCV, EBV, HHV8 and HTLV are direct carcinogens (5).

HPV contains a double-stranded DNA (dsDNA) genome that is approximately 8 kbps in length (6, 7). It causes almost all cervical, anal, genital, head and neck cancers and 30% of oropharyngeal cancers (8). EBV is also a DNA virus that has a dsDNA genome that is 175 kbps in length, and nearly 95% of healthy adults have asymptomatic infections with EBV (9). The effects of EBV infection vary by geographic location, but it mainly causes nasopharyngeal cancer (10), posttransplant lymphoproliferative disorders (PTLDs) (11), Burkitt lymphoma (BL) (12) and Hodgkin lymphoma (13). HBV contains a partial dsDNA genome that is approximately 3.2 kbps in length (14). HCV has a single-stranded RNA genome that is approximately 9.6 kbps in length (15). HBV and HCV can cause hepatitis with variable degrees of damage, which, more seriously, can lead to cirrhosis and hepatocellular carcinoma (16). Moreover, studies have shown that HCV and HBV infections cause pancreatic cancer (17). MCV is a DNA virus that is nearly 5.4 kbps in length (18). MCV often causes a relatively harmless infection that persists lifelong, although it can also cause serious skin cancers and Merkle cell carcinoma (MCC) (19, 20). It has also been reported that the probability of MCC in AIDS patients is 10 times that in ordinary patients (21). HTLV is approximately 9 kb in length (22). The retrovirus human T-cell lymphotropic virus type 1 (HTLV-1) has infected 10–20 million people, although most of them are asymptomatic (23). Some infected patients develop highly aggressive malignancies, such as adult T-cell leukemia/lymphoma and HTLV-1-associated myelopathy/tropical spastic palsy (24, 25). KSHV, also known as HHV8, is a DNA virus that often causes Kaposi’s sarcoma, which is a type of skin cancer (26). The entire genome of HHV8 is 14 kbps in length (27).

As research has progressed, the importance of viruses in the etiology of various cancers has become increasingly clear, and there are already several resources that collect and host relevant information on tumor viruses. For example, the NCBI Nucleotide database contains a large number of viral nucleotide sequences submitted by researchers (28). In addition, ViPR, which is a pathogenic virus database and analytical resource, contains more information about these viruses, including their sequences, genes, proteins, immune epitopes and so on, and provides some basic analytical tools, such as those for sequence alignment, phylogenetic inference and BLAST comparisons (29). However, there is no resource that is focused on cancer-associated viruses, and it is still difficult for researchers to obtain the reference genomes. Considering that the number of cancers caused by viral infections has increased dramatically, a complete database that could support research on the relationship between these viruses and diseases is urgently needed. Here, we introduce iCAV, which is an integrative database of cancer-associated viruses, with reference genomes and the related metadata for seven types of cancer-associated viruses. To ensure convenient usage of the database, all viruses are grouped by sample country and disease, and researchers can utilize the browsing functions to obtain the results of interest.

## Materials and Methods

### Data collection and processing

**Figure 1.**
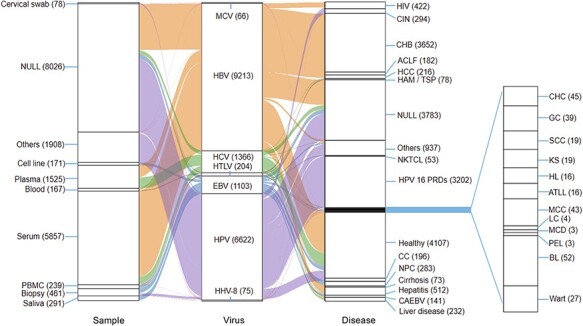
Relationships among samples, viruses and diseases. This figure shows mainly samples and diseases.

We searched for the nucleotide sequences of all seven viruses uploaded to the NCBI nucleotide database as of October 2020 using several carefully chosen keywords and then downloaded them (Table 1). To obtain the complete genome sequences, we first filtered the results by the range of genome length, which was defined as the known approximate length, and removed the sequences that only contain a portion of a genome (Table 1). Then, we extracted the relevant information for each virus, including the GenBank ID, definition, strain name, isolate name, geographic origin, sample type, and related phenotype. We also accessed the original study in which the virus sequences were published by searching for the PMID. For those genomes without PMIDs listed, we tried to obtain the relevant studies based on their reference titles. And then we searched the titles in PubMed database. At last, we took about 700 articles. All retrieved articles were carefully curated, the missing information, including countries, samples and phenotypes, were extracted from the original studies. The sample types were categorized by their source, such as plasma, serum, biopsy and cell line. We also carefully determined the country of the samples where the viruses were isolated. With regard to the phenotypes, we classified them into the specific disease or a healthy phenotype. Individuals who did not have any specific disease were defined as healthy (Figure 1). At last, detailed information, such as countries, samples and phenotypes, retrieved from NCBI nucleotide database and NCBI PubMed database were integrated, while the genomic sequences were also provided in iCAV (30).

**Table 1.** The keywords and length range of each virus

Virus	Keywords	Length
HTLV	Human T-cell lymphotropic virus OR HTLV OR Human T-lymphotropic virus	8000–10 000
HBV	Hepatitis B virus OR HBV	3000–3300
HCV	Hepacivirus C OR HCV	8900–10 000
MCV	Merkel cell polyomavirus OR MCV OR MCPyV	5000–5500
HPV	Human papillomavirus OR HPV	7000–10 000
HHV-8	Human gammaherpesvirus 8 KSHV Kaposi’s sarcoma- associated herpesvirus	130 000–140 000
EBV	Human herpesvirus 4 OR Human gamma- herpesvirus 4 OR EBV OR Epstein–Barr virus	160 000–180 000

### Construction of the website

The data we collected are stored in a MySQL database. The website was built using HTML, JavaScript and PHP, and several open source front-end libraries, such as jQuery and Bootstrap, were used to further modify the website (31). Then, the website was hosted on an Apache server. In addition, to ensure a smooth user experience, we tested the iCAV site on a variety of browsers, such as Google Chrome and Internet Explorer.

## Results

### Composition of the data in iCAV

In total, 18 649 reference genomes of seven types of viruses were collected, including 9213 HBV genome sequences, 6622 HPV genome sequences, 1366 HCV genome sequences, 1103 EBV genome sequences, 204 HTLV genome sequences, 75 HHV8 genome sequences and 66 MCV genome sequences (Figure 1). Our data were related to 87 phenotypes and 66 sample types, which originated from 143 countries worldwide, while the data types of each virus are presented in Figure 1, Tables 2 and 3. For example, in 9213 individuals infected with HBV, 3652 had chronic hepatitis B (CHB), 2575 were healthy, 462 had hepatitis, 175 had hepatocellular carcinoma (HCC) and 2349 had other diseases (Table 2). The sample types were 5410 serum samples, 119 blood samples, 772 plasma samples, 12 biopsy samples and 2892 other samples (Table 3). In 6622 individuals infected with HPV, 433 were healthy, 5973 had other disease, 196 had cervical cancer (CC), 19 had squamous-cell carcinoma (SCC) and 1 had genital cancer (GC) (Table 2). The sample types were 6372 other samples, 160 biopsies, 78 cervical swabs and 12 serum samples (Table 3).

**Table 2.** The phenotypes of each virus

Virus	Phenotypes	Count
HBV	CHB	3652
	Health	2575
	Hepatitis	462
	HCC	175
	Other diseases	2349
HPV	CC	196
	SCC	19
	GC	1
	Health	433
	Other diseases	5973
HCV	Hepatitis	50
	Chronic hepatitis C (CHC)	45
	HCC	41
	Health	794
	Other diseases	436
EBV	Nasopharyngeal cancer	283
	Natural-killer/T cell lymphoma (NKTCL)	53
	GC	26
	Hodgkin lymphoma	16
	Lung cancer (LC)	4
	Health	213
HTLV	HTLV-1-associated myelopathy/tropical spastic palsy	78
	Health	59
	Adult T-cell leukemia-lymphoma (ATLL)	16
	Other diseases	51
HHV8	Kaposi’s sarcoma (KS)	19
	Primary effusion lymphoma (PEL)	3
	Multicentric castleman disease (MCD)	3
	Other diseases	50
MCV	MCC	28
	Other diseases	5
	Health	33

**Table 3.** The samples of each virus

Virus	Samples	Count
HBV	Serum	5410
	Blood	119
	Plasma	772
	Biopsy	12
	Other samples	2892
HPV	Biopsy	160
	Other samples	6372
	Cervical swab	78
	Serum	12
HCV	Plasma	677
	Serum	434
	Cell lines	10
	Other samples	245
EBV	Saliva	291
	Other samples	371
	Biopsy	284
	Plasma	1
	Cell line	156
HTLV	Peripheral blood mononuclear cell (PBMC)	106
	Other samples	52
	Blood	45
	Plasma	1
HHV8	Other samples	56
	Biopsy	16
	Cell line	3
MCV	Other samples	54
	PBMC	5
	Biopsy	5
	Cell line	1
	Serum	1

### Usage and presentation in iCAV

**Figure 2.**
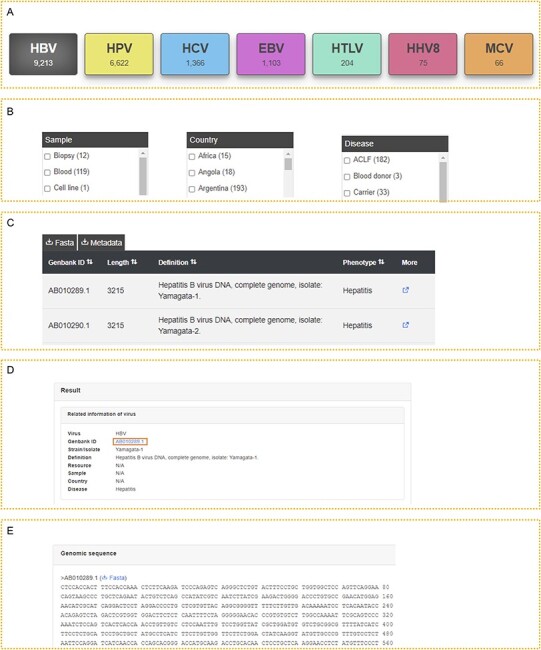
The usage of iCAV. (A–E) Five steps to use iCAV.

All data were classified by virus type, so it is convenient for users to access the corresponding records and relevant information for each type of virus (Figure 2A). We also listed the sample, country and disease on the left, which allows users to further filter the results (Figure 2B). After accessing the viruses of interest, users can download the genome sequences in FASTA format and the metadata (Figure 2C). Detailed information on the virus is provided if they click the ‘More’ link, including the GenBank ID, strain name, isolate name, definition, resource, sample, country and disease (Figure 2C–D). The complete genome sequence is also displayed (Figure 2E), and users can download it separately. Moreover, users can obtain all the data for their further analysis in the download page. We guarantee that we will not record any information about our visitors, including IP, private information and browsing histories.

## Discussion

These seven types of viruses, including EBV, HCV, HBV, HTLV, HHV8 and HPV, have been defined as Group 1 human carcinogens by the IARC, and MCV is classified as Group 2A (32). Among all cases of cancer caused by viral infections, the vast majority (>85%) occur in developing countries (33). Therefore, there is a growing urgency to study the relationship between viruses and cancers.

iCAV is the first database focused on cancer-associated viruses, and it provides users with the genome sequences and related phenotypes for seven human tumor viruses. By carefully collecting and integrating the metadata, iCAV can provide detailed information about the relationships between viruses and diseases that is easy for users to access. Our website provides a simple and straightforward interface for users to browse for the viruses of interest, and the results are clearly displayed and can be downloaded. In conclusion, iCAV is a convenient resource for researchers studying the relationships between virus genomic sequences and diseases and can improve research efficiency.

In this study, we collected the full and nearly full genome sequences of seven types of human cancer-associated viruses. The NCBI nucleotide database provides detailed sequence information, but the phenotypic information is missing. Therefore, we developed the iCAV database, which provides detailed phenotypic information to help us sort through large amounts of data to find the relevant information. Our goal is to integrate the available information about viruses and phenotypes. In the future, iCAV will be regularly maintained and updated every 2 years through surveying the lasted virus data of complete genome to provide more detailed and comprehensive information. We anticipate that the iCAV database will facilitate subsequent analyses by other researchers.
